# Fully flexible implantable neural probes for electrophysiology recording and controlled neurochemical modulation

**DOI:** 10.1038/s41378-024-00685-6

**Published:** 2024-06-27

**Authors:** Mohammad Hassan Malekoshoaraie, Bingchen Wu, Daniela D. Krahe, Zabir Ahmed, Stephen Pupa, Vishal Jain, Xinyan Tracy Cui, Maysamreza Chamanzar

**Affiliations:** 1 Electrical and Computer Engineering, Carnegie Mellon University, Pittsburgh, PA 15213 USA; 2 Bioengineering, University of Pittsburgh, Pittsburgh, PA 15260 USA; 3 Center for Neural Basis of Cognition, University of Pittsburgh and Carnegie Mellon University, Pittburgh, 15213 USA; 4 McGowan Institute for Regenerative Medicine, University of Pittsburgh, Pittsburgh, 15219 USA; 5 Carnegie Mellon Neuroscience Institute, Carnegie Mellon University, Pittsburgh, 15213 USA

**Keywords:** Electronic devices, Nanoparticles

## Abstract

Targeted delivery of neurochemicals and biomolecules for neuromodulation of brain activity is a powerful technique that, in addition to electrical recording and stimulation, enables a more thorough investigation of neural circuit dynamics. We have designed a novel, flexible, implantable neural probe capable of controlled, localized chemical stimulation and electrophysiology recording. The neural probe was implemented using planar micromachining processes on Parylene C, a mechanically flexible, biocompatible substrate. The probe shank features two large microelectrodes (chemical sites) for drug loading and sixteen small microelectrodes for electrophysiology recording to monitor neuronal response to drug release. To reduce the impedance while keeping the size of the microelectrodes small, poly(3,4-ethylenedioxythiophene) (PEDOT) was electrochemically coated on recording microelectrodes. In addition, PEDOT doped with mesoporous sulfonated silica nanoparticles (SNPs) was used on chemical sites to achieve controlled, electrically-actuated drug loading and releasing. Different neurotransmitters, including glutamate (Glu) and gamma-aminobutyric acid (GABA), were incorporated into the SNPs and electrically triggered to release repeatedly. An in vitro experiment was conducted to quantify the stimulated release profile by applying a sinusoidal voltage (0.5 V, 2 Hz). The flexible neural probe was implanted in the barrel cortex of the wild-type Sprague Dawley rats. As expected, due to their excitatory and inhibitory effects, Glu and GABA release caused a significant increase and decrease in neural activity, respectively, which was recorded by the recording microelectrodes. This novel flexible neural probe technology, combining on-demand chemical release and high-resolution electrophysiology recording, is an important addition to the neuroscience toolset used to dissect neural circuitry and investigate neural network connectivity.

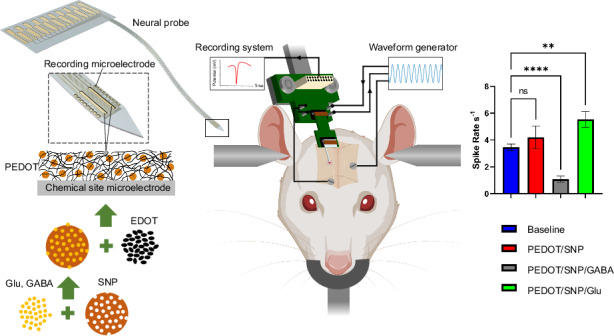

## Introduction

Implantable neural probes in the form of penetrating multi-electrode arrays have been widely used for electrophysiology recording in neuroscience research. With recent advancements in microfabrication technology, compact neural probes are now available for high-resolution electrophysiology recording^1–4^. Traditionally, these neural probes have been implemented on rigid substrates such as silicon. In order to minimize damage and tissue response and make these neural probes viable for chronic long-term interfacing with the brain, polymer substrates such as Parylene C^5,6^, Polyimide^7,8^, and SU8^9,10^ have also been used to implement flexible neural probes^11^. Other functionalities such as electrical and optical microstimulation^6,12^, as well as chemical sensing^13,14^ have also been added to these neural probes. Implantable neural devices for electrically controlled drug delivery have also attracted recent attention because of their ability to enable local neuromodulation. This new modality can be used for chemical modulation of neural activity to treat neurological disorders and study the role of neurochemicals in neural circuit dynamics. Localized drug delivery enables targeted therapy to the region of interest in the neural tissue, thereby enhancing the effectiveness of the treatment without the inherent side effects of systemic treatments^15,16^.

Fluidic delivery methods like pressurized injection, microcapillary tubes, or microfluidic channels have been traditionally used to deliver chemicals and drugs to the neural tissue. These methods are vulnerable to failures due to drug leakage, tip clogging, and reflux and usually require additional hardware, such as pumps and valves, which make the entire system complex and usually bulky. Moreover, incorporating fluidic channels into neural devices increases the overall size of the neural probe, thereby rendering it more invasive. Additionally, the pressure increase at the injection point might cause local edema^17–23^. Electrically controlled drug delivery can overcome some of these problems by offering highly localized and on-demand delivery of drugs^16,24^. In contrast to fluidic drug delivery devices, electrically controlled drug delivery releases only the drug of interest without the need for an artificial solvent. This dry delivery technique allows a larger amount of drug release with near-constant local pressure.

Conductive polymer coatings have been utilized in electrically controlled drug delivery. These polymers, doped with carriers that encase the chemicals of interest, are electrochemically deposited on the surface of electrodes. The most commonly used conducting polymers for this purpose include polypyrrole^25,26^ and poly(3,4-ethylenedioxythiophene) (PEDOT)^27–29^, into which various dopants can be incorporated. One limitation of this method is that the drug must be negatively charged. Another challenge is that the treatment or in vivo study needs to utilize a large enough dose of the drug to have an effect. Therefore, porous nanomaterials, like carbon nanotubes (CNTs)^30^ and graphene oxide nanosheets^31^, have been employed as drug carriers to enhance the loading capacity and tune the rate of drug release. Recently, silica-based sulfonated nanoparticles (SNPs)^24,32^ have also been utilized as conducting polymer dopants, which work as drug carriers for electrically controlled drug delivery. In addition to having substantial drug loading capacity and a variety of possible drug choices, SNPs can also insulate electroactive drugs from strong electric potential during loading and releasing, as they are non-conductive. As a result, SNPs improve the lifetime and stability of the loaded drug.

Current implantable neural devices for drug delivery lack sufficient integrated biosensors, hindering the optimization of treatments and performance evaluation. Incorporating electrophysiology recording electrodes into the design of a neural device for drug delivery is crucial to enable real-time monitoring of the behavior and activity of a larger population of neurons within the brain, providing greater detail and accuracy. This allows for a better assessment of drug regimen efficacy and neural circuit dynamics. Therefore, incorporating drug delivery electrodes in implantable neural devices can greatly enhance treatment outcomes and further the understanding of the underlying neural mechanisms.

Table 1 presents a comparison of neural probes that possess combined drug delivery and electrophysiology recording capabilities. Except for our prior research^16^, all these probes employ fluidic delivery techniques to deliver drugs. They vary in their number of channels, inlets, and outlets. While the majority can transport either a single chemical or a mixture at any given time, a few are equipped with independent fluidic systems, enabling them to deliver 2 distinct chemicals concurrently. They are also integrated with varying numbers of recording electrodes ranging from 2 to a maximum of 16. Most of these neural probes are constructed from a rigid silicon substrate. However, some also utilize flexible materials such as SU8, Parylene, PDMS, and Polyacrylate. In our previous study, we successfully demonstrated the feasibility of a novel electrically controlled delivery method^16^. This was implemented by modifying a rigid silicon Neuronexus probe, utilizing 4 electrode sites coated with PEDOT doped with carbon nanotubes as drug carriers, serving as a proof-of-concept for our approach. All 4 sites were stimulated at the same time in order to release sufficient drugs for detectable effects^16^.

**Table 1:** List of Implantable neural probes integrated with drug delivery and electrophysiology recording capabilities

	Author	Substrate	Footprint *w* × *t* (μm)	Number of Ephys recording electrodes	Impedance at 1 kHz (kΩ)	Drug delivery method	Number of chemical sites
1	Zhanhong Jeff Du^16^	Silicon	100 × 15 4 shanks	8	20	Electrically controlled delivery (PEDOT + CNT)	4 electrodes
2	Christopher M. Proctor^15^	SU-8/parylene C	80 × 54	2	N/A	Electrophoretic/ microfluidic ion pump	1 channel/5 outlets
3	Ximiao Wen^14^	PDMS	144 × 30 1–4 shanks	2 on each shank	21.4 ± 1.5	microfluidic channel	1 channel on each shank
4	Ane Altuna^9^	SU-8	90 × 55	8	>1000	microfluidic channel	2 channels
5	Hyogeun Shin^66^	Silicon	100 × 40	16	800	microfluidic channel	3 inlets/5 channels/1 outlet
6	Yousang Yoon^67^	Silicon	240 × 40	16	16 ± 2	microfluidic channel	1 inlet/10 channels
7	Pratik Rohatgi^68^	Silicon probe + catheter	165 × 165	16	N/A	microfluidic channel	1 channel
8	Johannes Gurke^69^	Polyacrylate/parylene	500 × 204	5	21.2 ± 6.4	microfluidic channel	2 channels
9	Anita Pongrácz^70^	Silicon	400 × 380	16	>400	microfluidic channel	2 channels
10	Sven Spieth^71^	Silicon	250 × 250 2 shanks	4 on each shank	N/A	microfluidic channel	1 on each shank

It has been demonstrated that rigid neural implants can cause more significant damage to the neural tissue^33–35^. The glial scarring and tissue response depend on the dimensions and stiffness of the neural implants^36,37^. The substantial mechanical mismatch between soft neural tissue and rigid neural implants leads to tissue damage^38^. Furthermore, tissue damage is intensified by brain micromovements that come from sources like cardiac rhythm, fluctuations in respiratory pressure, and head movements^39–41^. On the other hand, flexible neural implants have been shown to mitigate brain tissue damage. Quantitative analysis of inflammatory biomarkers reveals that silicon probes elicit the most intense foreign body reaction compared to soft polymer probes^42^.

In this study, we engineered fully flexible neural implants with chemical modulation and electrophysiology recording capabilities. The neural probe was implemented using planar microfabrication processes on a mechanically flexible and biocompatible substrate, Parylene C. Two types of electrodes were microfabricated on the probe shank, including 16 small microelectrodes for electrophysiology recording to monitor neuronal response to drug release, and 2 large microelectrodes, for loading sufficient drugs. To better distinguish between the two types of microelectrodes in this paper, we refer to the larger microelectrode as a ”chemical site”. To reduce the impedance while keeping the size of the microelectrodes small, PEDOT/PSS was electrochemically deposited onto the recording microelectrodes. In addition, PEDOT doped with SNPs was used on chemical sites to achieve controlled drug delivery. Two neurotransmitters, glutamate (Glu) and gamma-aminobutyric acid (GABA), were incorporated into the SNPs. The impedance of all microelectrodes before and after polymer coating was measured using electrochemical impedance spectroscopy (EIS) measurement. The drug release profile was characterized in vitro by applying a sinusoidal voltage (0.5 V, 2 Hz) and quantifying the amount of chemical release as a function of the stimulation cycle. Finally, the flexible neural probe was implanted in the barrel cortex of the wild-type Sprague Dawley rats using a tungsten wire shuttle glued with Polyethylene glycol (PEG), and the neural activity was compared before and after the electrically stimulated neurotransmitter release.

## Device design and architecture

**Fig. 1:**
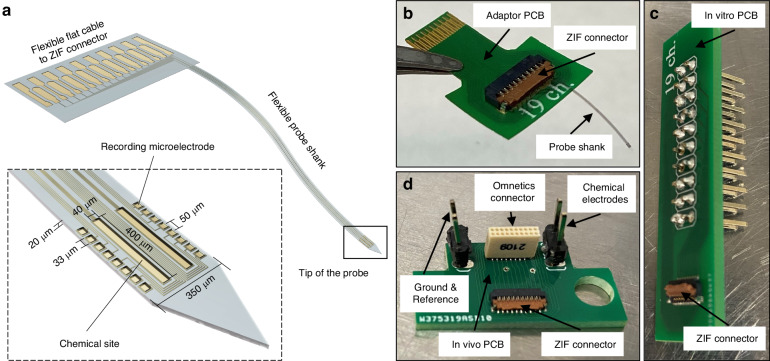
Neural probe design and architecture. **a** The schematic of the designed fully flexible neural probe. The inset figure indicates that the neural probe tip consists of an array of recording microelectrodes and 2 chemical sites. **b** Packaged neural probe with adaptor PCB. It has a ZIF connector at one end and a flat cable at the other end, both with 0.3 mm pitch size. **c** The in vitro PCB has the same ZIF connector in addition to 2.54 mm pitch size pin headers to connect the adaptor PCB to the modular potentiostat/galvanostat and waveform generator. **d** In vivo PCB equipped with the same ZIF connector and also a 16-channel Omnetics Connector to connect the adaptor PCB to the head stage of the electrophysiological recording system

Figure 1 depicts the schematic of the designed neural probe. It is fully flexible as it is made of Parylene C, a flexible polymer with Young’s modulus of about 2.75 GPa. Additionally, Parylene C is biocompatible and exhibits high degradation resistance to long biological tissue exposure^43^. It is used in FDA approved clinical implants. The footprint of the neural probe consists of a thickness of 20 μm and a width of 350 μm. This neural probe is designed to implant into different brain regions of Sprague Dawley rats and record the neural activities corresponding to chemical stimulation. The length of the probe shank is designed to be 10 mm so that it can cover cortical laminae and, at the same time, reach deep brain nuclei. It is also routed to be at the side of the probe backend to provide more space in the wafer layout, enabling efficient fabrication of multiple probes close to each other on the silicon (Si) wafer. The backend of the probe consists of a 0.3 mm pitch flat cable.

The inset of Fig. 1a offers a closer look at the tip of the neural probe that includes 2 identical large chemical sites, each with dimensions of 40 μm × 400 μm, extending along the length of the shank. This design provides sufficient capacity for chemical loading. To monitor the effect of chemical stimulation, we designed 16 recoding microelectrodes, which are positioned adjacent to the chemical sites with dimensions of 33 μm × 33 μm (~1000 μm^2^) and a pitch size of 50 μm. These recording microelectrodes allow for high-resolution electrophysiology recording while keeping the impedance of the microelectrodes low enough to achieve a high signal-to-noise ratio. Overall, the tip of the neural probe includes a total of 18 microelectrodes. The electrical traces connecting the recording microelectrodes to the backend of the probe have a width of 4 μm and are spaced 4 μm apart from each other. Since a larger electrical signal is applied to the 2 chemical sites through their dedicated interconnect traces, they are designed to have a width of 10 μm to ensure the reliability of these 2 traces. This is a more conservative choice compared to the traces that serve the recording microelectrodes.

Figure 1b, c illustrates the different sets of printed circuit boards (PCB; PCBWay, China) that we designed in order to package the neural probes for use in different experiments. One PCB was made for the in vitro and one for the in vivo experiment, as well as one adaptor PCB for each probe to bridge the flat cable at the backend of the probe to the two former PCBs. The adaptor PCB (Fig. 1b) features a zero-insertion force (ZIF; Hirose Electric Co., Japan) connector at one end and a flat cable at the other end, both with 0.3 mm pitch size. Due to the lack of commercially available 18-channel ZIF connectors compatible with our design, we have opted to use a 19-channel ZIF connector instead, and as a result, one non-connected bond pad is added to the design of the flat cable of the probe backend. Since the neural probe is very thin (20 μm), the backend is supported by a 125 μm thick Kapton tape with a 40 μm thick silicone adhesive (Bertech Inc., Torrance, CA) to increase the thickness of the probe backend for inserting into the ZIF connector. The in vitro PCB (Fig. 1c) has the same ZIF connector as the adaptor PCB in addition to 2.54 mm pitch size pin headers to connect the adaptor PCB to the modular potentiostat/galvanostat and waveform generator. The in vivo PCB (Fig. 1d) is also equipped with the same ZIF connector as the adaptor and in vitro PCBs, along with a 16-channel Omnetics connector (Omnetics Connector Corporation, Minneapolis, MN) in order to connect the adaptor PCB to the headstage of the electrophysiological recording system. It also includes two pin headers, each one belonging to one chemical site to connect it to the waveform generator providing the stimulation signal. Finally, it features two other pin headers for ground and reference, which connect to the skull screws during the in vivo experiments.

## Results

### Fabrication

**Fig. 2:**
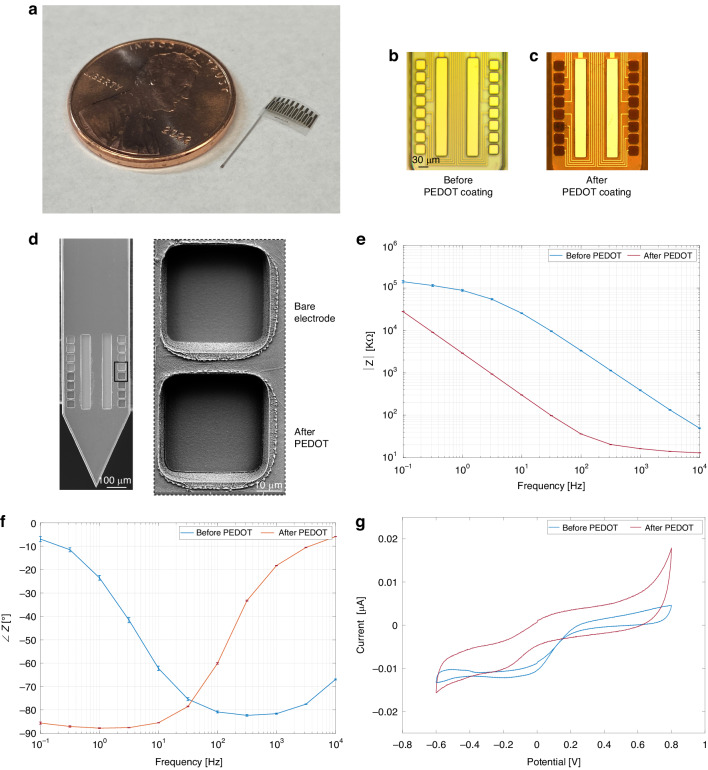
Characterization and post-fabrication modification of microelectrodes. **a** Released neural probe next to a penny for size comparison. **b** Tip of the neural probes, which includes 2 chemical sites and 16 recording microelectrodes, after the fabrication process and ready for post-processing. **c** Tip of the neural probes with PEDOT:PSS-coated recording microelectrodes. **d** SEM image of the probe shank with microelectrodes. The inset provides a higher magnification view of two electrodes, showcasing the difference before and after PEDOT coating. The rough morphology of the PEDOT coating is evident. **e** Bode plot, the impedance magnitude (mean ± SE, *n* *=* 128 microelectrodes) versus frequency before and after PEDOT:PSS coating. The impedance decreases significantly after PEDOT:PSS deposition. **f** Bode plot, the phase (mean ± SE, *n* *=* 128 microelectrodes) versus frequency before and after PEDOT:PSS coating. The microelectrodes after PEDOT:PSS deposition are capacitive in the low-frequency range and more resistive at higher frequencies. **g** CV plot for microelectrodes both before and after PEDOT coating

We have developed a scalable microfabrication process combined with post-fabrication drug loading to implement fully flexible neural probes with an integrated drug delivery system. Figure 2a, shows the fabricated fully flexible neural probe next to a penny for size comparison. The neural probe had a shank of 10 mm long without any curvature. The fabrication method was based on planar micromachining of thin polymer films on a 4-in. Si wafer. Parylene C polymer was used as the lower and upper insulation. For the electric layer, which contains the microelectrodes, bond pads, and traces, a metal stack that included gold (Au) sandwiched between two Platinum (Pt) layers was used. In addition to the excellent biocompatibility property of Pt, it also adheres well to Parylene C^44^. Au was chosen because it has the highest electrical conductivity (*σ* = 4.25 × 10^7^ S/m)^45^ among biocompatible materials. The microelectrodes and bond pads were opened through the Parylene C upper insulation layer, and the outlines of each device were defined using Oxygen plasma (Fig. 2b). Following fabrication, neural probes were released, and then they needed to undergo an annealing process to enhance the Parylene C chain entanglement and release stress^46^. In the materials and methods section, each fabrication step is thoroughly explained with detailed elaboration.

### Characterization and post-fabrication modification

The neural probes were packaged with adaptor PCBs to be characterized and prepared for in vitro and in vivo experiments. Post-fabrication processes were necessary to prepare the microelectrode surfaces and to load chemicals. The biocompatible conductive polymer poly(3,4-ethylenedioxythiophene) polystyrene sulfonate (PEDOT:PSS) was electrochemically deposited onto the recording microelectrode surfaces. This enhanced the signal-to-noise ratio of the recording by increasing the surface area of microelectrodes, thus reducing the impedance. In Fig. 2c, the fully covered coating of PEDOT:PSS on all recording microelectrodes is clearly visible. Figure 2d displays the SEM image of the probe shank with electrodes. Only the bottom eight electrodes are coated with PEDOT for comparison with the top uncoated electrodes. The inset provides a higher magnification view of two electrodes, showcasing the difference before and after PEDOT coating. The rough morphology of the PEDOT coating is evident. Additionally, biocompatible SNPs, which are capable of carrying chemicals, were doped into the conductive polymer PEDOT electrodeposited on chemical sites as a drug delivery platform^24^.

To ensure optimal performance and quality of the recording microelectrodes after PEDOT:PSS deposition, their impedance was evaluated at varying frequencies using electrochemical impedance spectroscopy (EIS). Figure 2e and f show the Bode plots across frequencies of interest (0.1–10 kHz) for recording microelectrodes (Pt/Au/Pt) with a dimension of 33 μm × 33 μm (~1000 μm^2^) before and after electrochemical deposition of PEDOT:PSS. The average ± standard error (SE) of impedance magnitude and phase for 128 recording microelectrodes were used in the Bode plots. Encouragingly, the recording microelectrodes demonstrated a higher than 93.75% yield. The impedance at 1 kHz was used for comparison, as neuronal spiking has a characteristic frequency band around that frequency. Before PEDOT:PSS coating, the average impedance was 387.64 ± 9.04 kΩ whereas it decreased to 16.18 ± 0.14 kΩ (~22.95 fold) after polymer deposition. The reduction in impedance is an inherent trait of PEDOT:PSS coatings, attributed to their substantial effective surface area^47^. The corresponding phase plot of the impedance shows that the PEDOT:PSS coated microelectrodes were capacitive in the low-frequency range (0.1–10 Hz) and more resistive at higher frequencies (1–10 kHz). Figure 2g presents the cyclic voltammetry (CV) plot for recording microelectrodes both before and after PEDOT coating. Evaluating the area beneath the cathodic curve, the charge storage capacity (CSC) of the microelectrodes prior to PEDOT coating was 0.13 mC/cm^2^. After the PEDOT coating, this value increased to 0.5 mC/cm^2^.

To measure the potential interference of the stimulation signal on recording microelectrodes, we designed an experiment in which the stimulation sinusoidal voltage (2 Hz, 0.5 V) was applied to one of the chemical sites in PBS, and the resulting signal was recorded through recording microelectrodes using the recording system (Fig. S1). The amplitude of the recorded stimulation signal varied between approximately 80 and 250 μV, depending on the distance from the chemical site (Fig. S2). Although the recorded voltage was minimal, to prevent interference, we decided to only consider neural activity right after the end of the stimulation period.

### In vitro

**Fig. 3:**
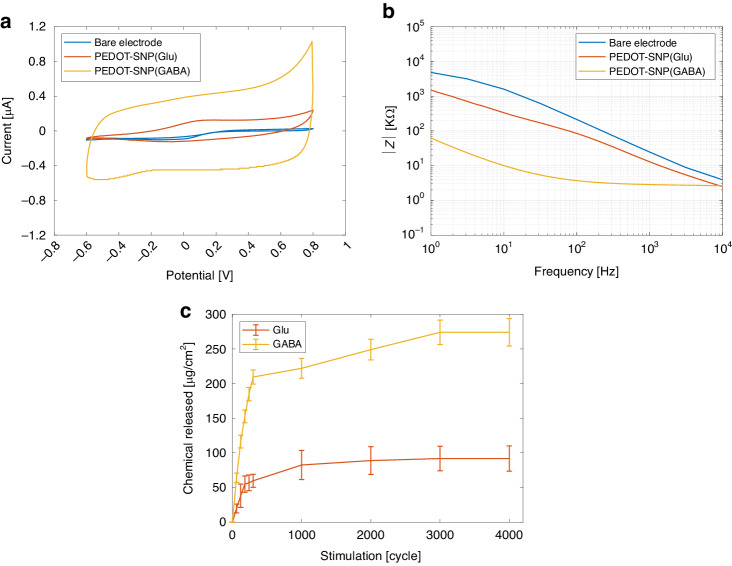
In vitro electrochemical characterization of PEDOT-SNP film doped with Glu and GABA on Pt pad chemical sites with a geometric area of 16,000 µm^2^. **a** CV characterization of PEDOT-SNP(Glu) film and PEDOT-SNP(GABA) film. **b** EIS characterizations of PEDOT-SNP(Glu) film and PEDOT-SNP(GABA) film. **c** In vitro drug release profile determination of Glu and GABA from chemical electrodes. Release profile of Glu released from PEDOT-SNP(Glu) film and GABA released from PEDOT-SNP(GABA) film. Three neural probes were used for each of GABA and Glu groups

The electrochemical properties of the PEDOT/SNP drug film were characterized by CV and EIS measurements. In comparison to the bare Pt chemical sites, PEDOT/SNP(GABA) and PEDOT/SNP(Glu) showed significantly increased CSC based on the CV curves (Fig. 3a) and significantly decreased impedance across the full frequency range examined (Fig. 3b). The cathodic CSC of PEDOT/SNP(GABA) and PEDOT/SNP(Glu) respectively are 1.90 ± 0.93 mC/cm^2^ and 1.31 ± 0.37 mC/cm^2^.

Drug release profile determination occurred by applying the stimulation signal (2 Hz, ± 0.5 V) to the chemical sites and measuring the concentration of the released drug in PBS by fluorometry (see Materials and methods for details). The PEDOT film is reduced by the applied waveform pulses, resulting in structure opening, thereby allowing PBS to penetrate the spaces where the SNPs are caged^24^. The drug diffuses out of the nanoparticle pores into the PBS, which is flushed back out after each stimulation. The release profiles recorded for both GABA and Glu (Fig. 3c) suggest that a majority of the drug was released from the polymerized film within the first 1000 stimulations. Based on the results collected from the 16000 μm^2^ chemical sites, the average mass of total drug stored and released after 4000 stimulations was close to 90 μg/cm^2^ and 275 μg/cm^2^ of chemical site surface for Glu and GABA, respectively. These in vitro results show that a notable amount of each drug (Glu and GABA) can be loaded onto the chemical sites, which can be subsequently released in a very controlled way. Before 300 stimulations, the data was collected every 60 stimulations where there was no data point between 300 and 1000 stimulations. The data collection frequency caused a sharp change in the in vitro release profile.

### In vivo

Coating chemical sites for in vivo experiments was completed using the same methods described in the materials and methods section for in vitro release characterization. On 1 of 2 chemical sites, a negative control was created by polymerizing EDOT with non-drug-loaded SNPs. SNPs were loaded with either GABA (initial loading solution: 10 mg SNPs, 1300 mg GABA, 1 mL DI water) or glutamate (10 mg SNPs, 700 mg glutamate, 1 mL DI water) and coated onto the surface of the second chemical site.

**Fig. 4:**
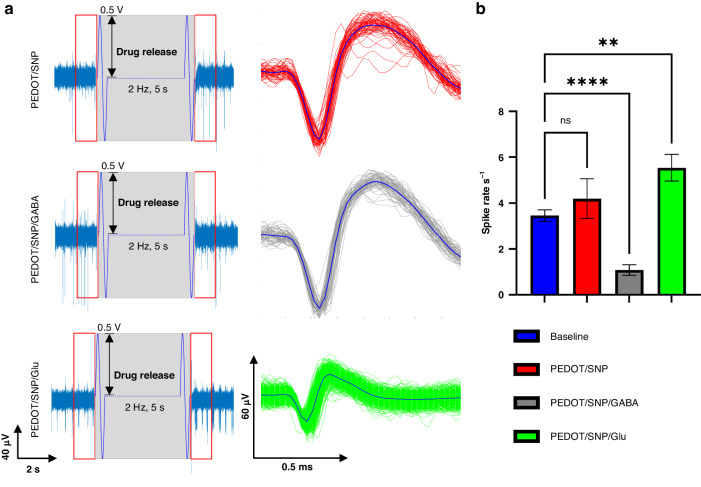
Electrophysiology data analysis. **a** Representative stream trace data and sorted single unit waveforms for all three neural probes loading conditions. The grey box indicates when sinusoidal pulses were applied to trigger the drug release. The red boxes indicate time windows where the number of spikes was counted for analysis. **b** Summary results from all conditions. As expected, compared to baseline (blue), Glu release caused a significant increase in spike rates (green), GABA release caused a significant decrease in spike rates (grey), and non-loaded PEDOT/SNP coating showed no changes in spike rates (red). Mean ± SE, *n* *=* 36, 3 neural probes in 3 individual animals for PEDOT/SNP. *n* *=* 62, 3 neural probes in 3 individual animals for PEDOT/SNP/GABA, *n* *=* 92, 3 neural probes in 3 individual animals for PEDOT/SNP/Glu. One-way ANOVA, Dunnett’s test, **p *<* 0.005, ****p *<* 0.0001. individual animals for PEDOT/SNP/Glu. One-way ANOVA, Dunnett’s test, **p *<* 0.005, ****p *<* 0.0001

Figure 4a shows the representative recording time series (left column) and sorted waveforms (right column) for all three experimental conditions. The red box in the time series data shows a 1 s time bin used to quantify the spike rates before and after drug release stimulation. The spike rates were quantified and shown in Fig. 4b. Stimulating the PEDOT/SNP without drug load showed no effects on spike rates compared to no stimulation, while stimulating chemical sites with the PEDOT/SNP(GABA) coating showed a significant decrease in spike rates (*p* < 0.005), and stimulating sites with the PEDOT/SNP(Glu) coating showed a significant increase in spike rates (*p* < 0.0001).

**Fig. 5:**
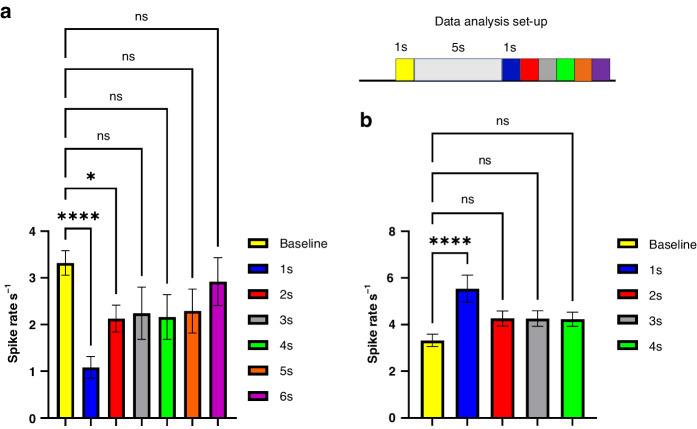
Temporal effects of the locally released neurotransmitters. Top schematic shows how data is grouped and analyzed. A 1 s time bin before and consecutive 1 s time bins after the release event were used to count the number of spikes. **a** Temporal effects of releasing GABA show inhibitory effects only sustained for 2 s, *n* *=* 92. **b** Temporal effects of releasing Glu show excitatory effects only sustained for 1 s, *n* *=* 92. One-way ANOVA, Dunnett’s test, *p *<* 0.05, ****p *<* 0.0001

To investigate the temporal resolution of drug release, a continued time bin analysis was used to quantify spike rates after stimulated drug release. The data analysis setup is shown on the top schematic bar in Fig. 5. A temporal response pattern is seen for PEDOT/SNP(GABA) and PEDOT/SNP(Glu). The spike rates returned to the baseline level 2 s after the GABA release (Fig. 5a), whereas it only took 1 s for spike rates to return to baseline after the Glu release (Fig. 5b). However, for both conditions, the effects of released neurochemicals were transient and reversible. The average signal-to-noise ratio (SNR) and single unit yield (SUY) of recording were used to quantify recording quality and are shown in Fig. S3a and Fig. S4b, respectively. The detailed calculation of SNR and SUY is described in the method section. The average SNR of all units included for analysis from each group is above 6. The overall average SUY from all groups is 20.40% ± 3.60%.

## Discussion

We demonstrate the design, fabrication, and packaging of multi-modal, fully flexible neural probes using scalable micromachining techniques for electrophysiology recording and controlled neurochemical modulation. Flexible neural probes for recording neural activity have been demonstrated before, and nanoparticle-doped PEDOT for electronically controlled drug delivery using a commercial silicon-based Neuronexus probe has been demonstrated in our previous work^16^. The novelty of this work lies in the integration of these pieces into a novel neural probe system with unique features. Our design offers 16 recording microelectrodes along with 2 chemical release sites specifically designed for the electrically controlled delivery mechanism. This allows simultaneous electrophysiology recording and on-demand chemical release for neural modulation. In contrast to our previous approach, which required the release of drugs from four small electrode sites simultaneously to achieve a detectable change in recorded neural activity, our new design leverages custom-designed chemical release sites with larger electrodes and increased chemical storage. In addition, instead of carbon nanotubes, which were used as drug carriers in our previous work, we used porous silica nanoparticles as a more versatile carrier in this work for the first time. Furthermore, unlike our previous work, which was implemented on a rigid neural probe, our current neural probe is fully flexible.

Our neural probe incorporates 16 recording microelectrodes and 2 chemical release sites, utilizing an electrically controlled delivery mechanism. As a result, our probe can deliver and release 2 independent chemicals simultaneously. Previous devices, as shown in Table 1, have offered microfluidic systems for drug delivery integrated with varying numbers of electrophysiology recording electrodes, ranging from 2 to a maximum of 16 channels. However, the fluidic drug delivery method has notable drawbacks and limitations, including drug leakage, tip clogging, reflux, and the need for complex and bulky systems^17–23^. They offered delivery of a maximum of 2 independent chemicals. It is worth emphasizing that our probe leverages a scalable microfabrication process, enabling a significant increase in the number and density of both recording microelectrodes and chemical release sites. Hence, our system can enhance the number of chemical deliveries without substantially increasing the size of the device. In contrast, adding an independent channel to a fluidic device would significantly increase its size.

The soft Parylene C substrate allows minimally invasive interfacing with neural tissue. Also, Parylene C is a biocompatible polymer that has been used safely in FDA approved biomedical implants. The neural probe has a long shank of 10 mm that can access different regions of rodent brains. The neural probes with flexible shanks can develop curvatures due to in-plane stress after the fabrication process, which creates difficulty in handling, assembly, and precision insertion. To avoid this issue, we employed several strategies. Firstly, we designed a symmetrical material stack to prevent the formation of residual stress. Secondly, we used e-beam evaporators for low-stress metal deposition^5^. Finally, we performed post-fabrication thermal annealing to release any residual stress that had formed during the manufacturing process^46^. Based on the previous reports, annealing also leads to thermally induced entanglement of the polymer chains and recrystallization to reduce stress and moisture permeation^48^.

Evaporated platinum films show less stress compared to those deposited by sputtering on Parylene C, as indicated by reduced wrinkling in the metal layer and less curvature in released devices^49^. This stress exacerbates adhesion failures and accelerates delamination in the long term. Additionally, we improved the adhesion of evaporated platinum film on Parylene by oxygen plasma treatment to clean and roughen the surface and induce hydrophilicity^50^.

To more effectively evaluate flexibility, we calculated the bending stiffness, an indicator of flexibility, for all neural probes with a polymer substrate listed in Table 1. These calculations are presented in Table S1. As observed, our neural probe, made from Parylene C, ranks second in flexibility after the PDMS probe. Notably, Young’s modulus of PDMS is three orders of magnitude smaller than other common polymers typically used in neural probes. In addition, none of these flexible probes offer 16 recording electrodes paired with 2 chemical sites capable of delivering 2 independent chemicals. Our probe features spatially multiplexed drug release capability, simultaneously delivering multiple independent drugs to targeted areas within the tissue. Also, the chemical sites are adequately sized to store sufficient drugs. The current design was deliberately conservative to demonstrate the viability of our approach in biology experiments. Utilizing a scalable microfabrication technique allows our device to have a more compact design, substantially reducing its size. We can achieve a reduced probe width by trading off the spatially multiplexed drug release capability and drug storage capacity for a design with fewer or smaller chemical sites. Also, the trace and spacing are currently 4 μm, which can be easily reduced to 1 μm or even less. Furthermore, in our present design, the traces circumnavigate the chemical sites to make recording electrodes as close as possible to the chemical sites to record from affected neurons. This can be streamlined in future iterations by routing the traces from just one side. We can also conduct multilayer microfabrication to stack traces and narrow the width.

Our neural probe exhibits the lowest thickness compared to other flexible devices offering both drug release and electrophysiology recording functionalities in Table 1. The next thinnest design after ours has a thickness of 30 µm^14^. Our choice of 20 μm thickness for the insulation layers (10 μm lower and 10 μm upper insulation) was a conservative compromise between the flexibility and reliability of the insulation layer. A 10 μm Parylene C film, once annealed, should be sufficient to prevent water/moisture penetration^50^.

Based on Table 1, the impedance of electrodes at 1 kHz starts from values above 1 MΩ and descends to the lowest reported value of 16 ± 2 kΩ, associated with Pt black-coated electrodes. Our electrodes, coated with PEDOT:PSS, also demonstrate a low impedance, measuring at 16.18 ± 0.14 kΩ. PEDOT:PSS coating effectively lowers the impedance by 22.95-folds. Achieving a low impedance is important for high signal-to-noise ratio neural recording, especially for small microelectrodes^47^. Figure S4 demonstrates the images and EIS measurements of the recording microelectrodes before and after in vivo experiment. The PEDOT:PSS coatings remained stable following mechanical interactions with tissue. EIS measurements show a slight increase in impedance at high frequency after surgery which both indicate the adhesion of PEDOT:PSS to the microelectrodes was sufficiently robust during the in vivo experiment.

Drug-loaded SNPs were electropolymerized into a PEDOT film. This mechanism of drug release allows us to control the onset and duration of release since the surrounding solution can only enter the PEDOT/SNP film and flush the stored drug from the SNP pores while it is being electrically stimulated. We characterized the release profile from the fabricated chemical sites and, consistent with our previous results, the majority of drug release appears to take place within the initial 1000 stimulation cycles, with a significant reduction in the amount of releasable drug after this point. Approximately 100 µg/cm^2^ GABA and 300 µg/cm^2^ Glu can be released from the films. Other molecules, including different neurotransmitters, modulators, and pharmacological agents such as receptor agonists and antagonists, could be loaded onto the electrode surface and released into the brain in a controlled way using this method of electrical actuation. The size of our drug-release electrodes can be optimized to store different amounts of biochemicals and to locally release the molecules at the target locations of interest in the future.

In our previous work^24^, we confirmed that the passive release of Glu from the electrode in the absence of the electrical signal was negligible. To minimize the passive release of the drug that was loosely incorporated into the conducting polymer layer, we washed the SNP prior to electropolymerization to effectively eliminate the majority of free Glu present in the supernatant. Subsequent experiments were done to verify the absence of detectable diffusion of Glu from the washed coating. The result demonstrated that our drug delivery system did not passively release detectable amounts of drug without the trigger, both before and after stimulation.

Implantation methods are another crucial factor affecting the in vivo performance of flexible neural probes. A recently published review paper discusses available methods for insertion of flexible neural probes^51^. A variety of insertion strategies have been utilized for minimum invasive insertion of flexible neural probes, including using removable shuttles, temporary stiffening agents, surface guiding devices, stiffness changing polymer substrate or coating, engineered cross-section structure of neural probe shanks, etc. All available methods differ in their surgical difficulties and targeting accuracy, but the lack of available parameters on different approaches makes quantitative comparison of immune system responses using different implantation methods difficult. Because of the high accuracy and moderate surgical difficulty of removable shuttles, we adapted the tungsten wire removable shuttle following the established methods reported in the literature^52^.

Here, we demonstrated a “dry” drug delivery method for in vivo neuromodulations. The PEDOT/SNP coating enables on-demand and local GABA/Glu delivery that causes transient and reversible modulation of neuronal activities. Compared to a previous electrophoretic drug delivery system^15^, the PEDOT/SNP coating approach obviates the need to incorporate microfluidic channels on the neural probe shanks that can significantly reduce the footprints of such neural probes. This is beneficial for chronic tissue-device integration, as indicated in previous research^53–55^. Also, sing PEDOT/SNP coating is a facile method that can be readily applied to any multi-electrode array neural probe, as it does not require pre-fabrication design changes. We demonstrated a reduction of neural spontaneous firing rates after local delivery of GABA, as expected from the inhibitory effects of GABA^56^. The firing rate recovered to the baseline rate within 2 s after the local delivery of GABA. Following the local delivery of Glu, we observed a transient increase in spontaneous neuron firing rate that faded away only 1 s after delivering Glu. The slower temporal response could be because of slower dynamics of metabotropic receptors GABA_b_^57,58^_._ Glu is known to have high complexity and integration in the reaction of astrocytes to neuronal activity. Literature also suggests that glutamatergic fluxes exceed GABAergic fluxes in rat cortex, so we speculate this could be the reason for the temporal difference in clearance time for GABA versus Glu^59–62^.

It was also important to make sure that the drug release stimulus itself did not affect neuronal firing by using the PEDOT/SNP control. We specifically chose to use a slow sine wave stimulus to minimize the chance of neuronal activation by electrical effects so as not to confound the main chemical stimulation effect. Other waveforms and durations might be explored to maximize efficiency and speed^63^. It is not clear, based on the current experimental paradigm, whether shorter than 5 s of stimulation would produce the same effect. Due to the stimulation artifact, we cannot quantify the neural activity during the 5 s stimulation train. It is plausible that the inhibition/excitation effect occurred shortly after the stimulus onset. Additional effort may be needed to eliminate the stimulus artifact further and enable recording during the stimulus.

Ten periods of sine wave were sufficient to release meaningful amounts of neurotransmitters (100 µg/cm^2^ GABA and 300 µg/cm^2^ Glu) that induce a significant change in neuroactivity in vivo. Based on the in vitro release profile, in theory, we should be able to produce the same neuromodulation effect up to 100 times from the same electrode coating.

In this work, we have successfully demonstrated proof-of-concept acute application of the proposed technology. However, to enable the chronic application of these neural probes, some aspects of the design should be improved: (1) Design of the backend connectors: The design of the adaptor PCBs and connectors can be miniaturized for chronic applications. (2) Drug loading capacity: Without devising a reloading mechanism, the PEDOT/SNP/drug coatings need to be tailored toward specific applications and the experimental timeline. (3) Tissue inflammation response: The implantation using a thick tungsten shuttle causes acute damage to the neural tissue, which can be partially healed after explanation of the shuttle^64^. The implantation method can be optimized to minimize tissue damage. In our current design, the probe dimensions are gauged toward robustness and handling for acute experiments as proof of concept. The chronic application and detailed tissue inflammation study would be more meaningful when the probes are further optimized with thinner insulation and narrower shanks for better tissue integration.

Combined with high spatial and temporal resolution electrophysiology recording, this novel chemical stimulation tool can find applications in a variety of acute neural circuit studies. Compared to technologies that have an external reservoir and fluid delivery, this tool is currently limited in drug load. Future research will aim to increase the drug loading capacity and optimize the release stimulus while exploring drug refill and/or recycle mechanisms to extend the lifetime of device functionality.

## Conclusion

We have developed novel flexible neural implants capable of controlled localized chemical stimulation and electrophysiology recording. The neural probes were implemented using planar micromachining processes on Parylene C, a mechanically flexible biocompatible substrate. The neural probe featured two types of microelectrodes: 2 large chemical sites for drug loading and 16 small microelectrodes for recording electrophysiology signals to monitor neuronal response to the drug release. PEDOT:PSS electrodeposition reduced the impedance by nearly 22.95-folds while keeping the size of the microelectrodes small to achieve higher fidelity of neuronal signals. In addition, PEDOT doped with SNPs on chemical sites was used to achieve controlled drug loading and releasing. Excitatory Glu and inhibitory GABA were incorporated into the SNPs and electrically triggered to release repeatedly. The in vitro experiment demonstrated on-demand chemical release by applying a sinusoidal electric voltage. Approximately 100 µg/cm^2^ of GABA and 300 µg/cm^2^ of Glu can be released from the films upon stimulation. The flexible neural probe was implanted in the barrel cortex of wild-type Sprague Dawley rats using a tungsten wire shuttle. Glu and GABA release caused a significant increase and decrease in neural activity, respectively, recorded by the recording microelectrodes. This novel flexible neural probe technology combining on-demand chemical release and high-resolution electrophysiology recording is an important addition to the neuroscience toolset for studying the electro-chemical dynamics of neural circuitry and investigating neural network connectivity.

## Materials and methods

### Fabrication

**Fig. 6:**
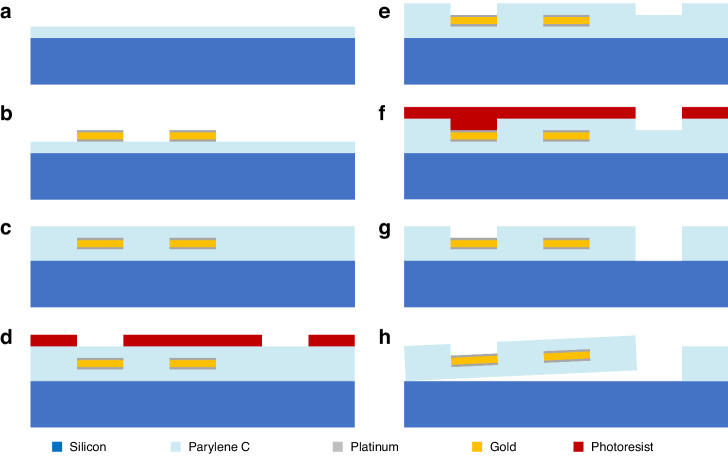
Scalable microfabrication process of the neural probe. **a** After the cleaning process, 10 µm Parylene C is deposited on a 4-in. Si wafer as the lower insulation layer. **b** A metal stack, including Au sandwiched between two Pt layers, is deposited on the lower insulation layer using metal evaporation following the liftoff process. **c** For upper insulation, 10 µm Parylene C is deposited again. **d** Microelectrodes, bond pads, and outlines are patterned on 15 μm spin-coated photoresist as an etch mask. **e** The upper insulation layer is etched by oxygen plasma. Then the photoresist mask is removed using Acetone, IPA, and DI water. **f** Outlines are patterned on 20 μm spin-coated photoresist as an etch mask. **g** The lower insulation layer is etched by oxygen plasma to etch the remaining outlines. Then the photoresist mask is removed using Acetone, IPA, and DI water. **h** Finally, while the wafer is soaking in the DI water, neural probes are released by gentle poking with a sharp tweezer

Figure 6 provides a detailed illustration of each microfabrication step in the process. A 4-in. Si wafer was cleaned with acetone, isopropyl alcohol (IPA), and deionized (DI) water consecutively and then dehydrated for 30 min at 95 °C. A 10 μm Parylene C (Specialty Coating Systems, Indianapolis, IN) layer was deposited on Si substrate as the lower insulation (Fig. 6a) using a Chemical Vapor Deposition (CVD) system (Specialty Coating Systems, Indianapolis, IN). To verify the thickness of the lower insulation, the coated Parylene C film was measured with a spectroscopic reflectometer (Nanospec 210XP, Nanometrics Inc. Milpitas, CA).

The lift-off process was used to define the electric layer on lower insulation Parylene C (Fig. 6b). An image reversal photoresist (AZ5214, Integrated Micro Materials, Argyle, TX) with a thickness of 1.4 μm was spin-coated onto the Parylene C layer (step 1: 10 s, 500 rpm, step 2: 60 s, 4,000 rpm). This will serve as an effective lift-off mask due to its negatively sloped sidewalls. The coated layer was then prebaked at 95° C for 4 min on a hotplate to evaporate the solvent, followed by UV exposure to pattern the electric layer, using a mask aligner (MA6, Suss MicroTec Co., Germany). A reversal bake was then carried out at the critical temperature of 120° C on a hotplate for 2 min to activate the special crosslinking agent present in the image reversal photoresist at the UV-exposed area. As a result, this area became almost insoluble in the developer and was no longer a light-sensitive substance, while the unexposed areas still behaved like a normal unexposed positive photoresist. By flood UV exposure (200 mJ/cm^2^) of the entire surface, the unexposed area could be dissolved in the standard developer (AZ400k [1:4]; Integrated Micro Materials, Argyle, TX). Before deposition of the metals, the lower insulation surface was cleaned and activated using oxygen plasma in a reactive ion etcher (RIE; Phantom II RIE, Trion Technology, Clearwater, FL) at 100 W and 100 mTorr for 30 s. A Pt (10 nm: 3 Å/s)/Au (100 nm: 5 Å/s)/Pt (10 nm: 3 Å/s) metal stack was deposited on the patterned photoresist mask using an electron-beam evaporator (PRO Line PVD 75, Kurt J. Lesker, Pittsburgh, PA). To remove unwanted metals, the wafer was soaked in acetone for 3 h, followed by flashing by a syringe. Finally, the wafer was washed consecutively with acetone, IPA, and DI water.

After the lift-off process and before the deposition of the upper insulation Parylene C layer, oxygen plasma was used to clean and roughen the surface of the lower insulation layer and metal stack (using the same process mentioned earlier); the surface was then dehydrated at 95 °C for 30 min. Subsequently, the 10 μm upper insulation Parylene C layer was deposited (Fig. 6c), the same as the lower one that was described previously.

To finalize the fabrication process, it is necessary to etch the upper and lower insulation layers and define the microelectrodes, bond pads, and device outlines. This was accomplished through a two-step etching process. Firstly, the upper insulation layer was etched to expose the microelectrodes and bond pads and to define half of the depth of the neural probe outlines. In the second step, the remaining outlines were removed from the lower insulation layer, effectively separating the devices from one another.

For the first step to form an etch mask that defined the microelectrodes, bond pads, and outlines of devices, a 15 μm thick positive photoresist (AZ4620, Integrated Micro Materials, Argyle, TX) was spin-coated onto the upper insulation Parylene C layer (step 1: 5 s, 500 rpm, step 2: 60 s, 1200 rpm). The coated photoresist was then soft-baked on the hotplate for 12 min at 95 °C to ensure the complete removal of the solvent. After the soft bake, the photoresist was allowed to rehydrate for 60 min at room temperature. The mask aligner was employed to UV-expose the photoresist and pattern openings. Subsequently, the wafer was immersed in the developer solution (AZ400k [1:3]) to remove the UV-exposed photoresist, resulting in the formation of an etch mask. (Fig. 6d).

Before etching, the 15 μm depth of the trenches was measured using a surface profilometer (Tencor P-15 Stylus Profiler, KLA, Milpitas, California). The microelectrodes, bond pads, and half depth of the outline of devices were etched using oxygen plasma in RIE. Pulse etch cycles, including a 3-min etch phase (50 W, 50 mTorr, O2: 60 SCCM) and a 2-min rest phase, were used to etch the upper insulation Parylene C layer while preventing heat generation during the process. After etching, the thickness of the trenches was verified with the surface profilometer and was found to be approximately 15 µm deep, indicating that the etch rate of both the photoresist and the Parylene C were similar. The depth of the remaining outline was measured using a spectroscopic reflectometer and found to be approximately 10 µm. The shiny surface of the microelectrodes and bond pads was observed under an optical microscope to ensure that all the Parylene C was removed in those regions. Then, the remaining photoresist mask was consecutively washed in acetone, IPA, and DI water (Fig. 6e). The depth of the trenches was measured with a surface profilometer to be around 10 µm.

The second etch step required a new photoresist etch mask in order to define the remaining device outlines. To achieve this, a 10 μm photoresist layer was spin-coated (step 1: 10 s, 500 rpm and step 2: 60 s, 2000 rpm) onto the surface of the device with a soft-bake at 95 °C for 12 min. This process was repeated for a second 10 μm photoresist layer, resulting in a total thickness of 20 μm of photoresist on the surface. Since there were already 10 µm trenches and microelectrode openings from the previous step, the overall thickness of the photoresist layer was 30 µm. The same process in the first etch step was done to make the second photoresist etch mask. The thickness of the mask was measured using a surface profilometer, and the distance from the bottom of the trenches to the surface of the photoresist was found to be approximately 30 µm (Fig. 6f). Using the same etching conditions as described in the first step, the remaining Parylene C outlines were etched away. Microscopic examination confirmed that all Parylene C had been removed from the intended regions. Finally, the photoresist etch mask was removed using acetone, IPA, and DI water consecutively (Fig. 6g).

To use the neural probes for in vitro and in vivo experiments, they need to be released from the wafer and packaged with the adaptor PCBs. To release the probes, we benefited from the lack of strong adhesion between Parylene C and silicon. After the etching process was completed, the wafer was soaked in DI water, and the neural probes were gently peeled away from the Si substrate using a sharp tweezer.

For the annealing step, neural probes were sandwiched between two large Teflon blocks and then placed in a vacuum oven (1 Torr) at 200 °C for 48 h^48^.

### Characterization and post-fabrication modification

**Fig. 7:**
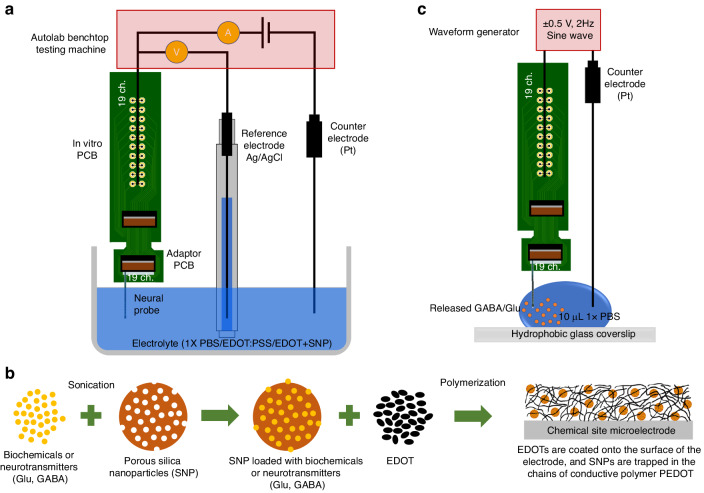
Characterization, post-fabrication modification, and in vitro setups. **a** Schematic representation electrochemical setup with three electrode configurations for CV and EIS characterization and PEDOT-(PSS, SNP) coating, which is conducted at room temperature. **b** Steps involved in the coating of Pt chemical sites with drug-loaded SNPs encased in PEDOT film. **c** Schematic representation of in vitro drug release experiment setup

We used a modular potentiostat/galvanostat benchtop testing machine (Autolab: Metrohm Co, Switzerland) to apply a 30 mV root mean square (RMS) sinusoidal signal to each channel, spanning a broad range of frequencies from 0.1 Hz to 10 kHz in order to generate an impedance spectrum. EIS measurements were performed at room temperature in 1× phosphate-buffered saline (PBS) electrolyte using a three-electrode configuration (Fig. 7a). An Ag/AgCl (3 M NaCl, MF-2052, BASi Co., West Lafayette, IN) was used as the reference electrode, and the counter electrode was a 7.5 cm long, 0.5 mm diameter, platinum wire (MW-1032, BASi Co., West Lafayette, IN). All of the electrochemical characterizations were conducted inside a Faraday cage.

The CV process was done by applying a square voltage signal from −0.6 V to 0.8 V, which is within the electrochemical window of water^65^ (the solution is neither oxidized nor reduced) for 15 cycles at a scan rate of 0.25 V/s.

To make the EDOT:PSS solution, 45 mg PSS was mixed with 40 mL of DI water for 1 h on a magnetic stirrer at room temperature. Then 40 μL of EDOT monomer was added to the solution and stirred for an additional hour. The solution was then stored in the refrigerator.

To electrodeposit PEDOT:PSS onto the recording microelectrodes, a modular potentiostat/galvanostat benchtop testing machine with a three-electrode configuration was employed (Fig. 7a), and a voltage of 0.85 V was applied to each recording microelectrode. To ensure full polymer film coverage on the surface and coating the same amount of polymer across all microelectrodes, we set two limitations: either the charge cut-off was 10^−9^ times the microelectrode area in μm^2^ unit (e.g., for 1000 μm^2^ microelectrodes the charge cut-off was 10^−6^ C) or the electrodeposition was carried out for a maximum of 300 s.

### In vitro

Mesoporous, sulfonate-modified silica nanoparticles (SNPs) were synthesized via a previously reported method^32^. Hexadecyl trimethylammonium bromide was first added to a mixture of triethanolamine, water, and ethanol to form the nanotubes, which eventually would constitute the pores. Next, tetraethyl orthosilicate and mercaptopropyl trimethoxysilane were added to form the thiolated silica nanoparticles around said pores. Surfactant within the pores was removed with an acid wash, and thiol groups were converted to sulfonate groups by the addition of hydrogen peroxide solution; this additional step allows the nanoparticles to be used as a conducting dopant. For the drug loading process onto chemical sites (Fig. 7b), 10 mg of SNPs were loaded with GABA or Glu via bath sonication (200 μl DI water, 1300 mg GABA, or 700 mg Glu) for 30 min to allow the collection of the drug inside the pores. Particles were collected via centrifugation and washed three times with DI water to remove excess GABA/Glu dissolved in the supernatant. Separately, a solution of 15 mM EDOT in DI water was prepared via probe sonication for 30 min. GABA/Glu loaded SNPs were sonicated with the EDOT solution for 5 min and then transferred to a glass cap to be used for electropolymerization. In the three-electrode configuration (Ag/AgCl reference, Pt foil counter) shown in Fig. 7a, the chemical sites surfaces were cleaned by submerging them in 0.05 M sulfuric acid and applying a CV staircase from 1.5 to −0.35 V until the current readout remained stable. After the cleaning process, chemical sites were submerged in EDOT/SNP solution, and an electrical current of 90 nA was applied to each of them for 1000 s to allow EDOT to polymerize and trap SNPs on the surface. PEDOT/SNP(GABA/Glu) coated chemical sites were gently rinsed with DI water to remove nonbonded residue and were stored dry. The electrochemical properties of the PEDOT/SNP drug film were characterized by CV and EIS measurements.

Drug release profile determination occurred using a sine waveform generator (DG1032Z, RIGOL Technologies INC, Portland, OR) (2 Hz, ± 0.5 V) into 10 μL of PBS using a Pt counter electrode, as shown in Fig. 7c. Stimulations occurred in bursts, with concentration sampled at 60, 120, 180, 240, 300, 1000, 2000, 3000, and 4000 stimulations. After each burst of stimulations, the PBS was collected for quantification, and a fresh 10 μL droplet of PBS was applied to mimic the drug diffusion in a real application. GABA concentration was determined using fluorescence intensity readings (excitation 550 nm, emission 590 nm, SepctraMax i3x, Molecular Devices, San Jose, CA). Glutamate concentrations were determined using a fluorescent glutamate detection kit (Amplex™ Red Glutamic Acid/Glutamate Oxidase Assay Kit, Invitrogen, Waltham, MA).

### In vivo

**Fig. 8:**
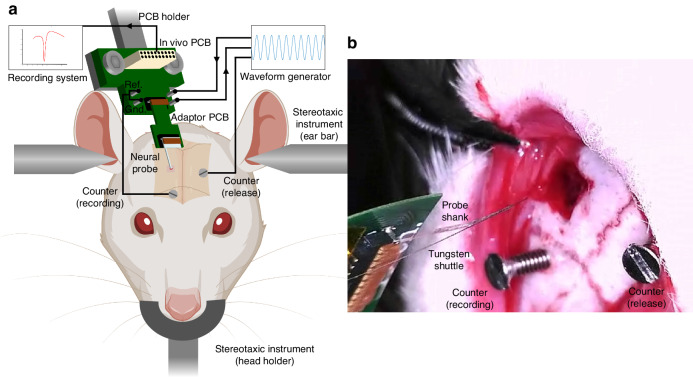
Implantation of neural probe in In vivo experiment. **a** Schematic of the setup for in vivo experiments on Sprague Dawley rats. **b** Location of craniotomy and counter screws. The recording microelectrodes array is implanted at a 15° angle. The neural probe shank is temporarily glued to a tungsten wire shuttle with PEG. The stainless-steel screw on the ipsilateral side was used as a counter for recording, and the one on the contralateral side was used as a counter for sinusoidal pulse delivery

All animal work was performed under the guidelines of the University of Pittsburgh Institutional Animal Care and Use Committee (IACUC). The approved protocol ID is 21099737. Sprague–Dawley rats were anesthetized under isoflurane (3%) and head-fixed in a Harvard Apparatus digital stereotaxic frame. Animal body temperature was maintained at 37 °C using an isothermal pad connected to a SomnoSuite system (Kent Scientific Corporation, Torrington, CT, USA). Heart rates were monitored using the SomnoSuite system as well. A schematic demonstration of the setup for the in vivo experiment on rats is shown in Fig. 8a. A customized 3D-printed holder was used to secure the custom-designed in vivo PCB, which is responsible for routing recorded signals to the head stage and delivering electrical actuation signals for stimulation. Omnetics connectors were used to interface with the TDT recording system (RX5, 16-channel Medusa amplifier, Tucker Davis Technologies (TDT), Alachua, FL). Two of the four PCB pins were shorted together and used as a combined counter/reference for recording. The remaining two pins were designed to connect to a waveform generator responsible for delivering the electrical actuation signal. Rats were anesthetized using isoflurane and head-fixed with ear bars on the stereotaxis. Two skull screws were carefully positioned above the right motor cortex and left visual cortex of the rat, as illustrated in Fig. 8b. A 1 mm × 1 mm window above the barrel cortex of the right hemisphere was opened using a motorized drill. The coordinates for the center of the barrel cortex were 2.5 mm posterior to Bregma and 5.5 mm lateral to the midline. The durotomy was performed with a #11 scalpel blade and a sharp tip tweezer. The packaged neural probe with an adaptor PCB was connected to the in vivo PCB, and the flexible shank was temporarily glued to a 50 µm diameter tungsten wire shuttle with 30% polyethylene glycol (PEG, MW 20KDa). The neural probe assembly was inserted into the cortex using a micromanipulator with a 15° angle and a depth of 1.5 mm beneath the cortical surface. Most of the microelectrodes were located in layer IV. The 15° angle is chosen because of the curvature near the lateral edge of the brain. This ensures that the implanted neural probes reach the accurate depth and avoid severing axonal connections between layers^33^. The 16 microelectrodes span ~300 µm vertically, covering tissue depth of 500 µm to 800 µm. The counter wire for electrophysiology recording was connected to the skull screw above the ipsilateral motor cortex, while the counter for the release trigger was a skull screw above the contralateral visual cortex. Utilizing separate counter electrodes for recording and drug release stimulation minimized stimulation artifact. Additionally, the in vivo PCB was designed to fully segregate the neural recording and electrical stimulation circuits, further reducing the potential for the stimulation artifact. The drug release trigger timing was synchronized by the RX5 recording processor. The sinusoid stimulation was triggered manually using the waveform generator, and the offset of stimulation was transmitted to the recording system using a customized TTL trigger. The electrical stimulation signal, consisting of a 2 Hz sine waveform with an amplitude of 0.5 V and a duration of 5 s, was generated using a waveform generator and delivered to the chemical sites to trigger the Glu/GABA release. The neural signal from recording microelectrodes was amplified using a 16-channel Medusa preamplifier and recorded with an RX5 processor at 25 kHz.

Neural signals were imported to MATLAB with custom scripts to remove stimulation artifacts. Plexon offline sorter (Plexon Inc Dallas, TX, USA) was used to identify single units. Raw data was filtered between 300 Hz and 10 kHz. Threshold crossing events were identified by using a fixed negative threshold value of 3.5 standard deviations. Plexon offline sorter (Plexon Inc Dallas, TX, USA) was used to identify single units. A 3D PCA feature space was used to identify waveform features, and the K-means clustering method was used to identify individual units. K-means were set up using an adaptive standard EM between 2 and 5. The SNR of sorted units was calculated, and units with an SNR above 4 were used for spike rate analysis. The SNR is calculated using the equation below:

$${\rm{SNR}}=\frac{{V}_{p2p}}{\sigma \left({V}_{\rm{noise}}\right)}$$

where *V*_p2p_ is peak to peak amplitude of single units, and *σ*(*V*_noise_) is the standard deviation of the noise floor.

The SUY is calculated using the equation below:

$${\rm{SUY}}=\frac{{N}_{\rm{active}}}{{{\rm{N}}}_{{\rm{total}}}}* 100 \%$$

Where *N*_active_ is the number of electrode sites that recorded single units, *N*_total_ is the total number of functional electrode sites per neural electrode. SUY is calculated for individual neural probes used in vivo and averaged together.

A customized MATLAB script was used to calculate SNR and SUY. Statistical analysis and results plotting were done using GraphPad Prism 10.0.0 and MATLAB 2019a. Data described in the text are Mean ± SE unless specified otherwise. Schemes were drawn with BioRender.

### Supplementary information

Supplementary Information
