# Supplementary material for: Fully flexible implantable neural probes for electrophysiology recording and controlled neurochemical modulation
Source: Microsyst Nanoeng. 2024 Jun 27;10:91. doi: 10.1038/s41378-024-00685-6 (PMC11211464; doi:10.1038/s41378-024-00685-6)
Supplement: Supplementary file 1 — Supplementary Information [file 41378_2024_685_MOESM1_ESM.docx]

**Supplementary Information: Fully flexible implantable neural probes for electrophysiology recording and controlled neurochemical modulation**


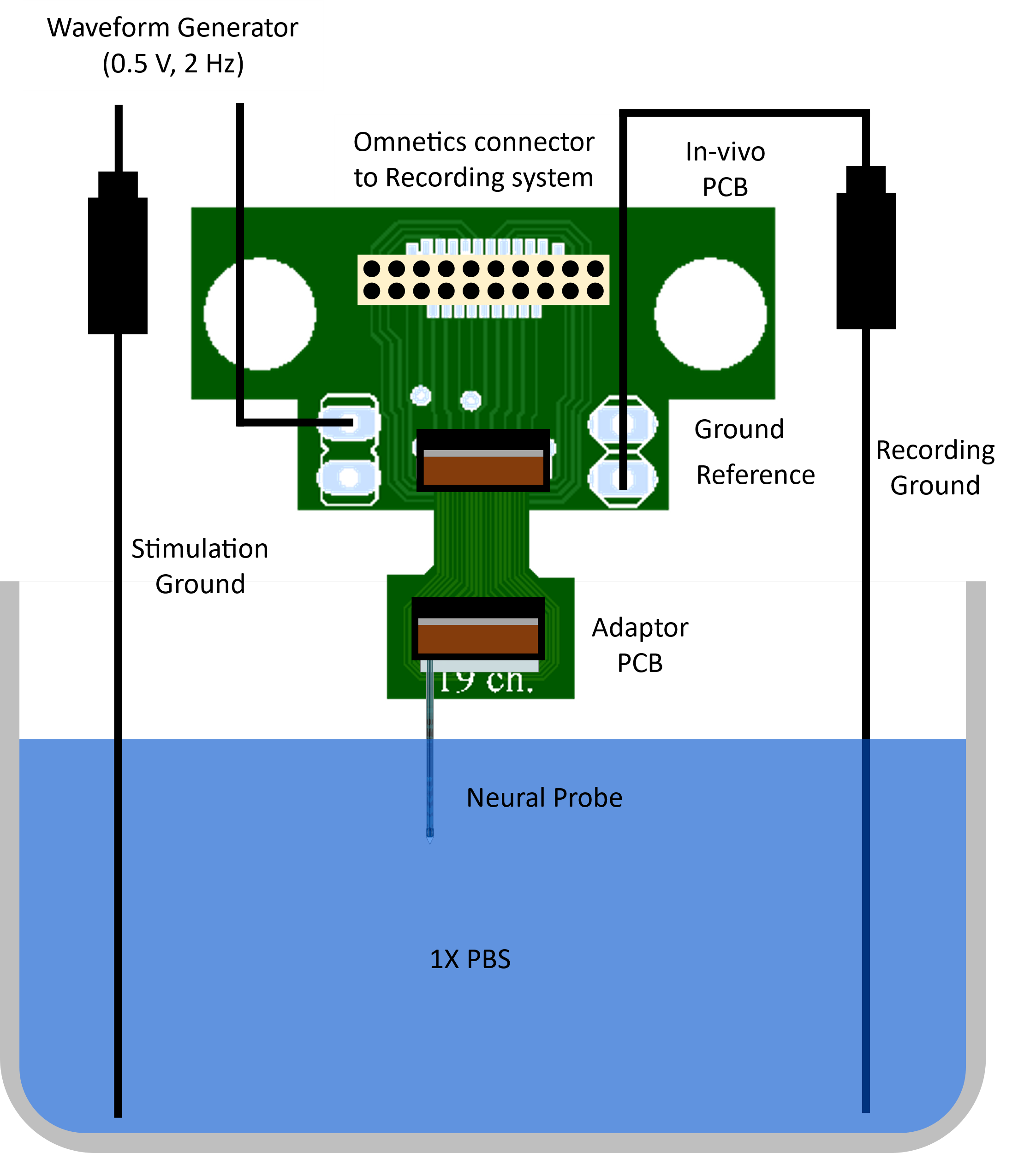


**Fig. S1:** To measure the potential interference of the stimulation voltage on recording microelectrodes, a synthetic experiment was designed, in which the stimulation sinusoidal voltage was applied to one of the chemical sites in PBS and the resulting signal was recorded through recording microelectrodes using the recording system. This figure shows the schematic of the synthetic experiment setup.


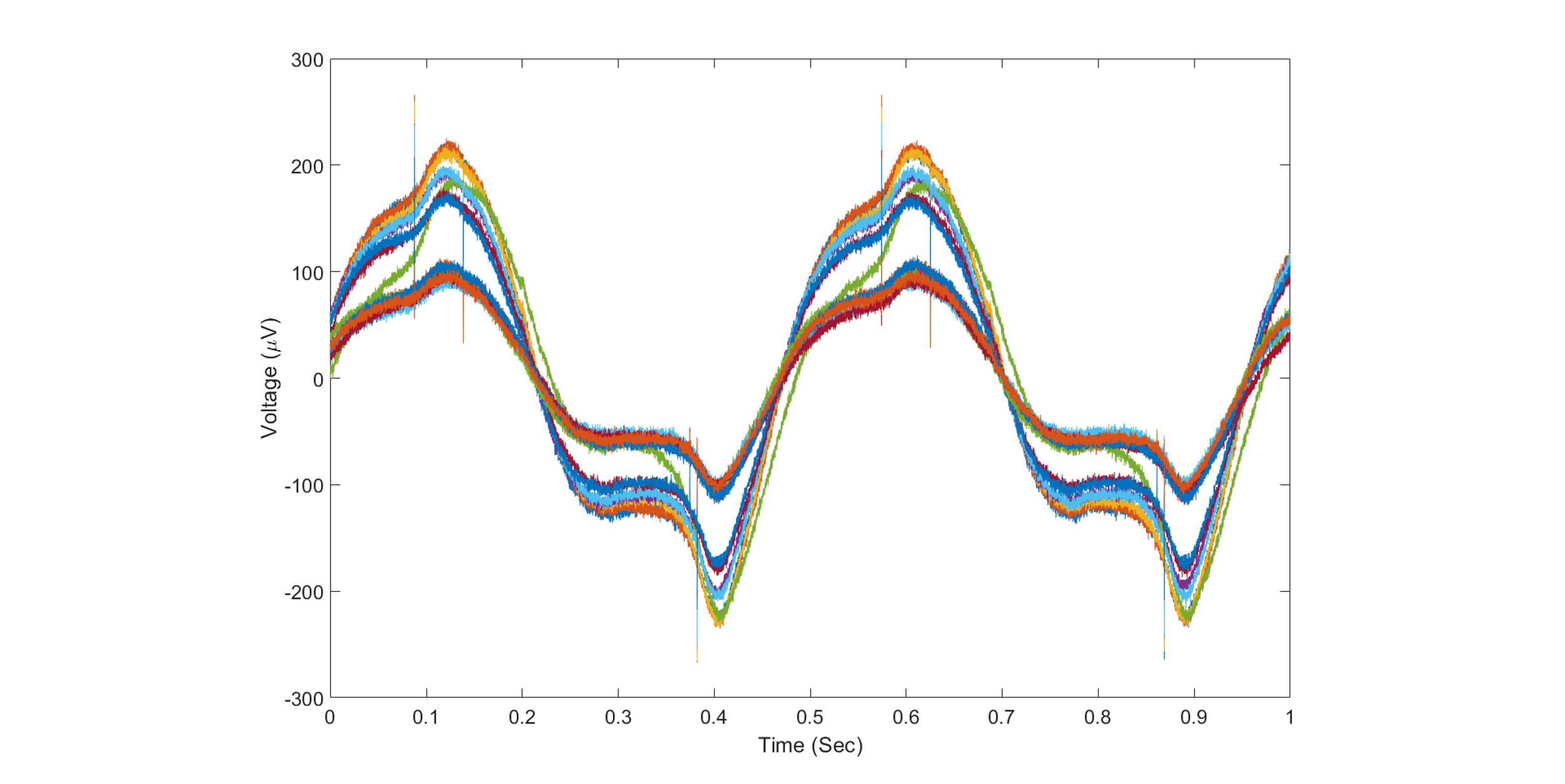


**Fig. S2:** The recorded stimulation signal by all recording microelectrodes in the synthetic experiment. In this experiment, only one of our large chemical sites was used for stimulation. Eight recording microelectrodes located near that chemical site detected a slightly higher signal, while eight more distant microelectrodes detected a weaker signal.


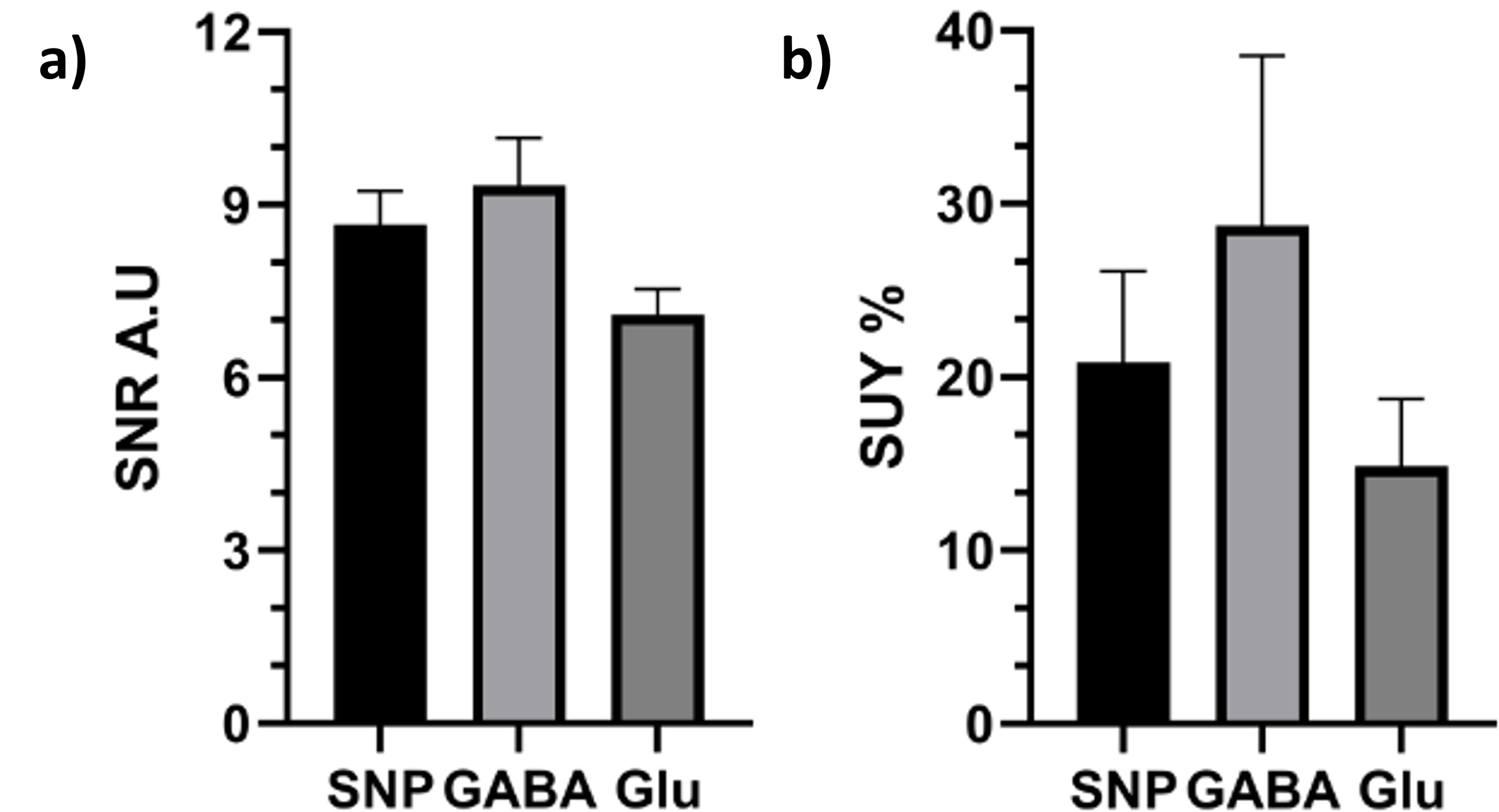


**Fig. S3: a,** Average SNR of all single units in each group used in in vivo analysis. **b,** Average SUY of all implanted neural probes in each animal group.


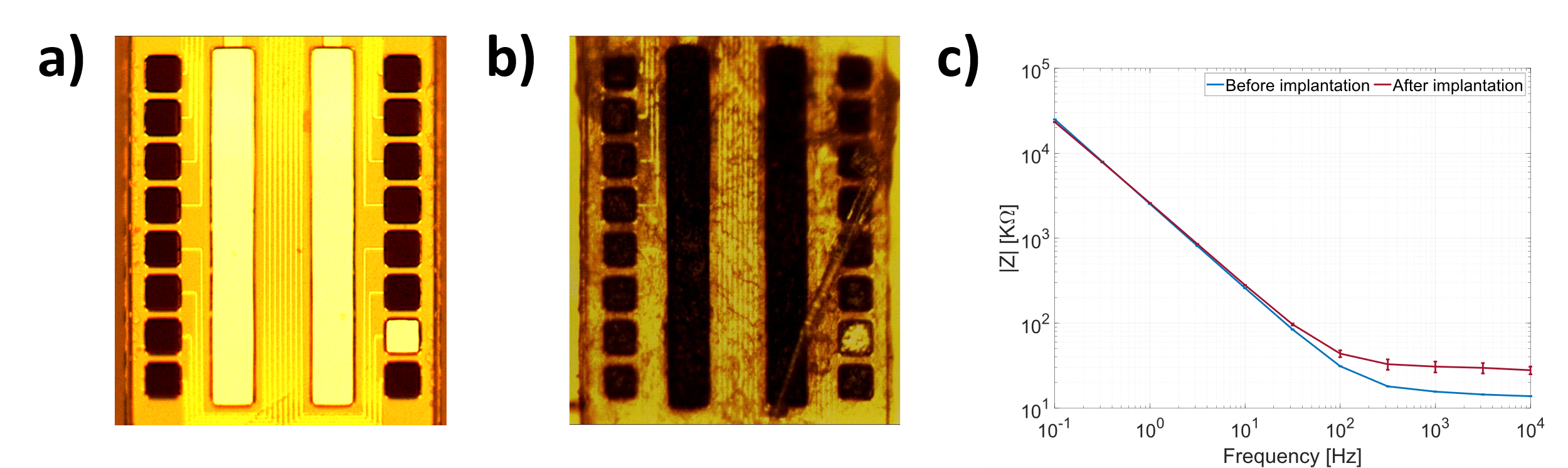


**Fig. S4: a,** Recording microelectrodes with PEDOT:PSS coating. The yield of electrodes was 93.75% and one of the electrodes was not working. **b,** Microelectrodes were observed after insertion and retraction, revealing that the PEDOT:PSS coating remained stable following mechanical interactions with tissue. **c,** EIS measurements before and after implantation, show a slight increase in impedance at high frequency after surgery. This indicates that the adhesion of coating was sufficiently robust during in vivo experiment.

**Bending Stiffness of the neural probe**

Flexibility is defined as the ability to bend easily under a given load. Bending stiffness refers to the resistance of a structure to bending under applied force. Thus, a beam with lower bending stiffness is more flexible. The bending stiffness of a beam, for example, depends on its material properties (like Young's modulus, which measures the stiffness of an elastic material) and its geometry (such as its cross-sectional shape and size). In technical terms, the bending stiffness of a beam is calculated as the product of Young's modulus (E) and the moment of inertia (I) which depends on the geometry of the cross-section of an object. Equation S1, shows the bending stiffness and the moment of inertia, where w is the width, and t is the thickness of the rectangular cross-section.

Bending stiffness $=EI=E\frac{wt^{3}}{12}$ Eq.S1

In Table S1, the bending stiffness of the probes with polymer substrate from table 1 is calculated.

| Author | Substrate [E (Mpa), t (μm)] | w (μm) | EI (N.μm^2^) |
| --- | --- | --- | --- |
| Christopher M. Proctor^15^ | SU-8 [2000,50] /Parylene C [2750,4] | 80 | 1667.8 |
| Ximiao Wen^14^ | PDMS [1.6,30] | 144 | 0.5 |
| Ane Altuna^9^ | SU-8 [2000,55] | 90 | 2495.6 |
| Johannes Gurke^69^ | Polyacrylate [2425,200]/Parylene C [2750,4] | 500 | 8083.4x10^2^ |
| **This study** | **Parylene C [2750,20]** | **350** | **641.7** |

**Table S1:** The substrate, dimensions and bending stiffness of flexible neural probes with electrophysiology recording and drug release capabilities.
